# Impact of the method of calculating 30-day readmission rate after hospitalization for heart failure. Data from the VancOuver CoastAL Acute Heart Failure (VOCAL-AHF) registry

**DOI:** 10.1093/ehjqcco/qcae026

**Published:** 2024-04-12

**Authors:** Samaneh Salimian, Sean A Virani, Thomas M Roston, Ren Jie Robert Yao, Ricky D Turgeon, Justin Ezekowitz, Nathaniel M Hawkins

**Affiliations:** Centre for Cardiovascular Innovation, Division of Cardiology, University of British Columbia, Vancouver V6T 2A1, Canada; Centre for Cardiovascular Innovation, Division of Cardiology, University of British Columbia, Vancouver V6T 2A1, Canada; Centre for Cardiovascular Innovation, Division of Cardiology, University of British Columbia, Vancouver V6T 2A1, Canada; Centre for Cardiovascular Innovation, Division of Cardiology, University of British Columbia, Vancouver V6T 2A1, Canada; Centre for Cardiovascular Innovation, Division of Cardiology, University of British Columbia, Vancouver V6T 2A1, Canada; Canadian Vigour Centre, University of Alberta, Edmonton, Alberta T6G 2E1, Canada; Centre for Cardiovascular Innovation, Division of Cardiology, University of British Columbia, Vancouver V6T 2A1, Canada

**Keywords:** Heart failure, Patient readmission, 30-day Readmission rate, Quality improvement, Standardized reporting

## Abstract

**Background:**

Thirty-day readmission rate after heart failure (HF) hospitalization is widely used to evaluate healthcare quality. Methodology may substantially influence estimated rates. We assessed the impact of different definitions on HF and all-cause readmission rates.

**Methods:**

Readmission rates were examined in 1835 patients discharged following HF hospitalization using 64 unique definitions derived from five methodological factors: (1) International Classification of Diseases-10 codes (broad vs. narrow), (2) index admission selection (single admission only first-in-year vs. random sample; or multiple admissions in year with vs. without 30-day blanking period), (3) variable denominator (number alive at discharge vs. number alive at 30 days), (4) follow-up period start (discharge date vs. day following discharge), and (5) annual reference period (calendar vs. fiscal). The impact of different factors was assessed using linear regression.

**Results:**

The calculated 30-day readmission rate for HF varied more than two-fold depending solely on the methodological approach (6.5–15.0%). All-cause admission rates exhibited similar variation (18.8–29.9%). The highest rates included all consecutive index admissions (HF 11.1–15.0%, all-cause 24.0–29.9%), and the lowest only one index admission per patient per year (HF 6.5–11.3%, all-cause 18.8–22.7%). When including multiple index admissions and compared with blanking the 30-day post-discharge, not blanking was associated with 2.3% higher readmission rates. Selecting a single admission per year with a first-in-year approach lowered readmission rates by 1.5%, while random-sampling admissions lowered estimates further by 5.2% (*P* < 0.001).

**Conclusion:**

Calculated 30-day readmission rates varied more than two-fold by altering methods. Transparent and consistent methods are needed to ensure reproducible and comparable reporting.

## Graphical Abstract

**Graphical Abstract:**
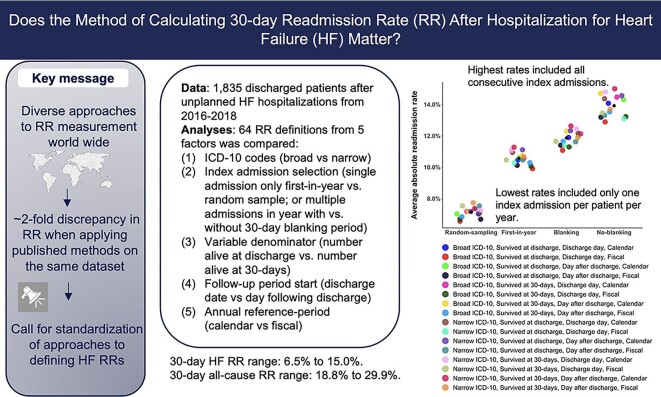

Key learning points What is already known?

- The 30-day readmission rate after a HF hospitalization is widely used to evaluate and compare healthcare quality and system performance.
- There are different methodological approaches to define the 30-day readmission rate, and no single accepted approach, which may influence the estimated rates. The combined impact of these methods is unknown.

What this study adds?

- The calculated readmission rate in the HF population is significantly influenced by various factors, including the index admission selection method, ICD-code definition, reference period, survival adjustment, and index day.
- It is important to accurately define and report each factor to ensure consistency in the calculation and comparison of readmission rates.
- Index admission selection should be performed with care, taking into account specific research questions or reporting purposes, to ensure that the rate comparisons are meaningful.

## Introduction

Heart failure (HF) hospitalizations are important to patients, providers, payers, and policymakers. Readmitted patients are a readily defined high-risk group with excess healthcare utilization and are thus a priority for healthcare interventions. The 30-day readmission rate is a widely adopted surrogate of healthcare quality used to evaluate and compare system performance regionally, nationally, and internationally.^1,2^ This apparently simple metric defines the proportion of patients readmitted (numerator) from the discharged population (denominator). However, methodological variations may impact estimates, including cohort selection, reference period, unit of analysis, index admission selection process, and consideration of mortality as a competing risk.

Most North American organizations analyse multiple episodes of care per patient, including the Agency for Healthcare Research and Quality (AHRQ),^3,4^ Centers for Medicare and Medicaid Services (CMS),^5^ and the Canadian Institute for Health Information.^6^ By contrast, the UK National HF Audit only examines a single episode per patient in the defined time frame.^7^ Within North American definitions, the AHRQ and UnitedHealth Group include hospitalizations as both index and readmission,^4^ whereas the CMS and 3M^TM^ Potentially Preventable Readmissions methodologies do not (Supplementary material online, Table S1).^5,8^ Such variations create challenges in comparing readmission rates across different populations, even after covariate adjustment.^9^ A recent systematic review identified marked global variation in 30-day readmission rates for patients with HF, which was not associated with the countries’ economic metrics, health care expenditure, or the competing risk of mortality.^10^ One potential explanation for this heterogeneity could be variations in how readmission rates are defined.

The combined impact of varying methodological factors on the estimated readmission rate has not previously been explored. We therefore estimated HF-specific readmission rates according to different definitions in the same dataset using a cohort of consecutive patients hospitalized with HF. Our objective is to raise awareness and provide the foundation for a standardized approach to defining HF readmissions.

## Methods

### Data and study population

The VancOuver CoastAL Acute Heart Failure (VOCAL-AHF) quality improvement audit assesses care in three hospitals in Vancouver, Canada. For this analysis, we included 1835 patients >18 years of age discharged after an unplanned hospitalization with a primary diagnosis of HF from 1 January 2016, through 31 December 2018, defined by International Classification of Diseases (ICD-10) codes. The day of final discharge after any inter-hospital transfers was considered the index day for the time to readmission calculation. Outcome ascertainment was conducted by linkage to the provincial administrative databases for hospitalizations (Discharge Abstract Database) and survival (Vital Statistics). The ethics review board of the University of British Columbia approved the study with a waiver of informed consent.

### Definitions

**Figure 1:**
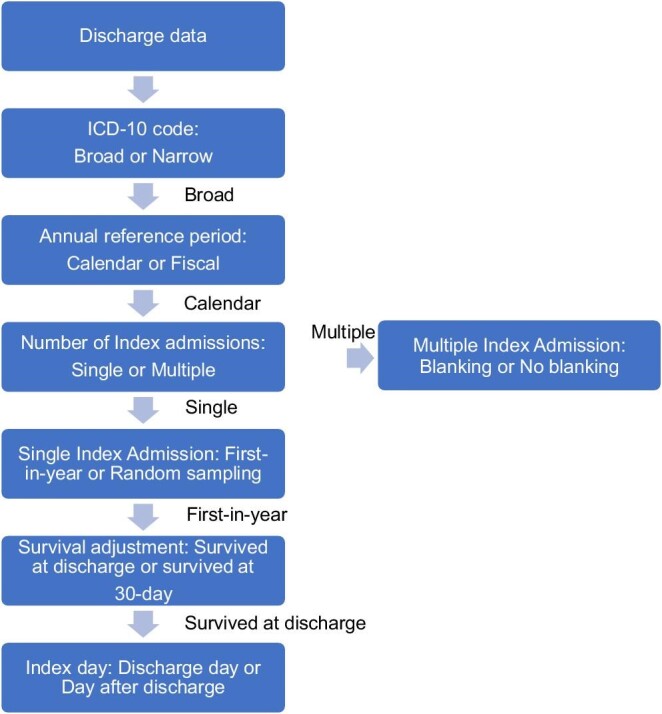
A flow diagram illustrating hypothetical factor combinations used in calculating readmission rates, assuming that one of the factors is selected to proceed.

We identified five distinct factors based on common approaches to defining readmission adopted by different healthcare organizations and research studies. Four factors were binary, and one (the index selection method) had four options. These were combined in different sequences to create 64 unique definitions of readmission rate for both HF-specific and all-cause readmissions. *Figure 1* illustrates the combinations as a flowchart. The following factors were defined (*Table 1*):

1. ICD-10 codes defining HF: Index hospitalizations were defined using either broad or narrow published code sets.^7,11,12^ The latter created a smaller population (*n* = 1663) with 172 fewer cases.
2. Index admission selection: This first classifies the inclusion of either single or multiple index admissions for each patient in a given year. Each then has a subcategory specific to that approach (*Figure 1*). The single index admission approach has two frequently used definitions: ‘first-in-year’ selects the initial admission in a given year (reference period) as the reference point for tracking readmissions within 30 days of discharge; ‘random sampling’ selects one admission randomly for patients with more than one hospital admission. When using a single index admission, the patient serves as the unit of analysis. The multiple index admission approach also has two possible definitions: ‘blanking’ considers only the initial index admission and any unplanned admissions that occur more than 30 days after the previous index admission as new index admissions; ‘no-blanking’ includes all unplanned admissions, regardless of the time elapsed since the previous index admission (Supplementary material online, Figure S1). When multiple index admissions are considered, the unit of analysis is the index admission. The equations for computing the rates through either of the index selection methods are presented in Supplementary material online, *Equation 1*.
3. 30-day survival: The denominator for the readmission rate includes individuals who survived to discharge or to 30 days after discharge.
4. Index day for counting time to readmission: Set as either the day of discharge or the day after discharge.
5. Annual reference period: Set as either a calendar or fiscal year.

**Table 1:** Overview of the binary definitions and categories for the primary factors impacting the readmission rate calculation

Categories	Factors
	**ICD-10 code**
Broad	I11.0, I25.5, I42.0, I42.6-I42.9, I43, I50.0, I50.1, I50.9
Narrow	I50.0, I50.1, I50.9
	**Number of index admissions**
Single index	Only one index admission is permitted for each patient in a year.
Multiple index	Multiple index admissions are permitted for each patient in a year.
	**Index admission selection method**
	*Single index admission*
First-in-year	Initial admission in a year served as the starting point for enumerating readmissions within 30 days of discharge from the initial index admission.
Random-sampling	For patients with more than one reported hospital admission, one admission is randomly selected, and readmissions within 30 days of discharge from the selected index admission are enumerated.
	*Multiple index admission*
Blanking	Initial and all unplanned admissions are considered as index admissions if they occur more than 30 days from the previous index admission.
No-blanking	Initial and all unplanned admissions are considered as index admission even if they occur less than 30 days from the previous index admission.
	**Survival adjustment**
Survived at discharge	The denominator for the readmission rate includes or excludes individuals who died within 30 days of discharge.
Survived at 30 days	
	**Index day**
Discharge day	The index day is the first day following a patient's initial hospitalization discharge, and it serves as the starting point for measuring the time until a potential readmission.
Day after discharge	
	**Reference period**
Calendar year	The reference year for reporting the readmission rates. January to December for Calendar year, and April to March for fiscal year.
Fiscal year	

To ensure consistent rate calculations with 30 days of follow-up, the final month of either the calendar year (December) or fiscal year (March) was excluded when randomly selecting index admissions for patients with multiple admissions.

### Statistical analysis

HF-specific and all-cause readmission rates were calculated for all 64 unique definitions using the same dataset. The readmission rates were averaged over 3 years to minimize random and secular variability. Seasonal variation in the proportion of index admissions over 3 years was shown graphically. Multiple linear regression was used to determine the impact of different factors on the readmission rate, which involved the use of dummy (in the case of the index admission selection method) and binary variables (other variables). An interaction term was included in the model between the index admission selection method and both the ICD-10 code and the reference period. The results of the statistical tests were considered significant at *P* < 0.05 (two-tailed). Statistical analysis was performed on R, version 4.2.1 (R Foundation for Statistical Computing).

## Results

Based on the five factors (four binary and one quaternary) and 64 unique definitions, for any particular factor, a range of readmission rates were demonstrated. For example, when defining readmissions based on a single admission selected randomly from the year (one of the four index admission selection options), the remaining four variables can also be varied (narrow vs. wide ICD-10 codes, variable denominator based on survival, index day discharge vs. post-discharge, and calendar vs. fiscal reference year).

### Heart failure readmission rates

**Figure 2:**
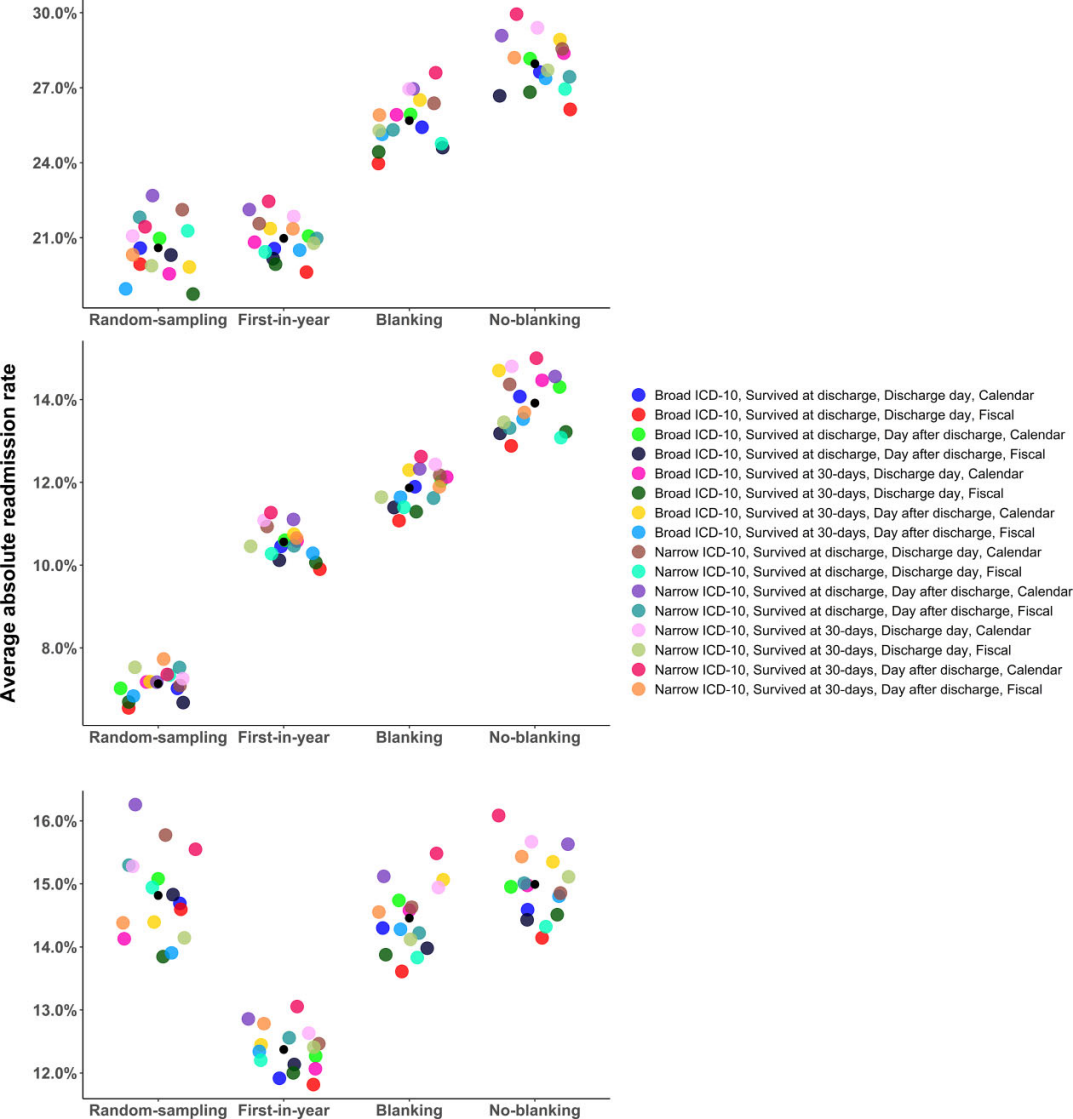
Absolute readmission rate by index admission method categories for all-cause, HF-readmissions, and non-HF readmissions (from top to bottom, respectively). Each colour on the plot represents a specific definition using the following factors: from left to right, the ICD-10 code (broad vs. narrow), 30-day survival (survived at discharge, survived at 30 days), index-day (discharge day, day after discharge), and annual reference period (calendar, fiscal). To enhance the representation of rate differences, the *y*-axis range is adjusted to optimize the visualization for each specific range. The points are scattered along the *x*-axis to prevent overlap. Supplementary material online, *Figure S2* presents the same figure with standardized *y*-axes for all 3 sub-figures.

The minimum and maximum absolute HF-specific readmission rates varied significantly from 6.5% to 15.0% respectively, the difference being 8.5% in absolute terms (*Figure 2*, Supplementary material online, *Table S2* and *Figure S2*).

A clear trend was apparent comparing the 64 unique readmission rates. Overall, 30-day readmission rates were highest when all consecutive (multiple) index admissions were included (11.1–15.0%). Conversely, readmission rates were lowest when considering only single index admissions per patient (6.5–11.3%). The significance of the index admission selection factor was validated using a linear regression model, which ranked the relative importance (association strength) of variables affecting the readmission rate in the following order: index selection method, annual reference period, ICD-10 codes defining HF, 30-day survival adjustment, and index day (*Table 2*).

**Table 2:** Association between cohort selection factors and HF or all-cause readmission rates using multiple linear regression

	HF admissions			All-cause admissions		
Factors	Change in readmission rate (%)	95% Confidence interval	*P* value	Change in readmission rate (%)	95% Confidence interval	*P* value
ICD-10 code (narrow vs. broad)	0.3	(+0.2, +0.4)	<0.001	0.9	(0.4, 1.4)	<0.001
Index admission (multiple readmissions with 30-day blanking)	Reference					
First-in-year	−1.5	(−1.6, −1.3)	<0.001	−5.0	(−5.6, −4.4)	<0.001
Random sampling	−5.2	(−5.3, −5.0)	<0.001	−5.7	(−6.3, −5.1)	<0.001
Multiple no-blanking	2.3	(+2.2, +2.5)	<0.001	2.3	(+1.7, +2.9)	<0.001
Survival (alive at 30 days vs. at discharge)	0.2	(+0.2, +0.3)	<0.001	0.1	(−0.1, +0.3)	<1
Index day (day after discharge vs. discharge day)	0.2	(+0.1, +0.3)	<0.001	0.5	(+0.3, +0.7)	<0.001
Reference period (fiscal vs. calendar)	−0.7	(−0.9, −0.6)	<0.001	−1.5	(2.0, −1.1)	<0.001
Interaction between index admission and ICD-10 code						
First-in-year	0.1	(0, +0.3)	<1	0.0	(−0.3, +0.8)	<1
Random sampling	0.2	(0, +0.4)	<0.05	0.6	(−0.1, +1.2)	<0.1
Multiple no-blanking	−0.1	(−0.2, +0.1)	<1	0.0	(−0.7, +0.6)	<1
Interaction between index admission and reference period						
First-in-year	0.2	(0, +0.4)	<1	0.5	(−0.6, +0.7)	<1
Random sampling	0.7	(+0.5, +0.9)	<0.001	0.7	(0, +1.3)	<0.05
Multiple no-blanking	−0.5	(−0.7, −0.3)	<0.001	−0.1	(−0.7, +0.6)	<1

#### Index admission selection

The method of defining index admission had a significant impact on the estimated readmission rates. When including multiple index admissions in each year, the absolute readmission rate ranged from 11.1% to 12.6% when blanking the initial 30 days post-discharge, compared to 12.9–15.0% with a multiple index no-blanking approach. In the adjusted model, no blanking compared with blanking for multiple index admissions resulted in a significantly 2.3% higher average readmission rate [95% CI (2.2, 2.5), *P* < 0.001]. When comparing these two approaches using a fiscal year reference period, this difference was attenuated but remained significant at 1.8%, suggesting that the effect of index admission selection on the readmission rate is influenced by the reference period (i.e. the significant interaction term presented in *Table 2*).

When considering only single index admissions, comparing a first-in-year vs. random sampling approach yielded absolute readmission rates ranging from 9.9% to 11.3% vs. 6.5% to 7.7%, respectively. In the adjusted model, a single index first-in-year approach had a 1.5% lower readmission rate, on average, compared with the multiple index blanking approach [95% CI (−1.6, −1.3), *P* < 0.001]. Moreover, a random sampling approach for single index admissions was associated with an even lower readmission rate by 5.2% compared with the multiple index blanking approach [95% CI (−5.3 to −5.0), *P* < 0.001]. The relationship between random sampling vs. multiple index had a significant interaction with both the reference period (fiscal vs. calendar year) and the ICD-10 codes used to define readmission (broad vs. narrow).

More severe left ventricular systolic dysfunction and a higher burden of comorbidities were observed in patients with multiple HF admissions when compared to those with a single admission during the year with no history of readmission (Supplementary material online, *Table S3*).

#### Other factors

The remaining factors had smaller associations with the estimated readmission rates. The readmission rate averaged 0.7% lower when comparing fiscal vs. calendar year reference periods [95% CI (−0.9, −0.6), *P* < 0.001]. A narrow vs. broad ICD-10 code definition of HF for both index cohort definition and readmissions was associated with 0.3% higher readmission rates [95% CI (0.2, 0.4), *P* < 0.001]. When the readmission rate denominator was measured at 30 days as opposed to discharge (i.e. excluding those who died in the early post-discharge period), the readmission rate was 0.2% higher [95% CI (0.2, 0.3), *P* < 0.001]. The readmission rate was also 0.2% higher when the day following discharge defined the start of the 30-day follow-up period [95% CI (0.1, 0.3), *P* < 0.001].

### All-cause readmission rates

The minimum and maximum absolute all-cause readmission rates varied from 18.8% to 29.9%, respectively (*Figure 2*). The absolute difference between these rates was 11.1% (Supplementary material online, *Table S4*). Similar to the HF-specific readmission rates, including all consecutive index admissions produced the highest 30-day readmission rates (ranging from 24.0% to 29.9%), whereas considering only single index admissions per patient led to the lowest rates (between 18.8% and 22.7%).

In the adjusted model, when compared with the multiple index blanking approach, both of the single index approaches had similarly lower readmission rates: first-in-year 5.0% [95% CI (−5.6, −4.4), *P* < 0.001] and random sampling 5.7% [95% CI (−6.3 to −5.1), *P* < 0.001] (*Table 2*). Unlike the adjusted model for HF-specific readmission rates, there was no significant difference in the all-cause readmission model when the readmission rate denominator was measured at 30 days as opposed to discharge.

## Discussion

Thirty-day hospital readmission (all-cause as well as disease-specific) is a ubiquitous measure of health system performance. Using the same dataset, we found marked variability when applying widely used approaches^13–17^ which more than doubled the estimated HF-specific readmission rate. Among these factors, the index admission definition had the greatest impact, followed by the reference period and ICD code definitions. For index admission selection, several factors significantly increased readmission rates, including multiple vs. single hospitalizations per patient, first-in-year sampling for single admissions per patient, and not blanking 30 days after the index for multiple admissions.

### Index admission selection

The readmission rate was highest when including all admissions as opposed to only one index admission per patient in the year (11.1–15.0% vs. 6.5–11.3% in HF-specific and 24.0–29.9% vs. 18.8–22.7% in all-cause readmissions). Both approaches are commonly used by international healthcare organizations and agencies: the US HCUP includes all admissions,^4^ whereas the NHS National HF Audit assesses single admissions.^7^ The inclusion of all admissions selects an older population with a higher risk of recurrent admissions who have worse left ventricular function and greater comorbidities (Supplementary material online, *Table S3*).^18^ A multiple-readmission definition is more inclusive and representative of resource utilization and therefore useful for managers and policymakers. Derived readmission rates may alternatively be employed, adjusting for covariates and comparing hospital-specific observed-to-expected ratios, but may be limited by administrative data in capturing covariates.^9^

Our results suggest the potential for significant selection bias and that random sampling is preferred if using only a single admission. Selecting the first as opposed to randomly sampling admissions throughout the year increased readmission rates (adjusted model, 3.7% in HF-specific and 0.7% in all-cause). The NHS National HF Audit uses first-in-year sampling.^7^ Both first-in-year and random sampling have been used extensively in observational research to examine trends in patient characteristics,^14^ HF management,^19,20^ hospitalizations,^15^ readmissions,^16^ and mortality.^17^ Both methods use per-person analysis and might be expected to generate similar population characteristics. However, secular trends in aetiology, comorbidity, and treatment may contribute. For example, over time, the proportion of HF with preserved ejection fraction is increasing, while the uptake of guideline-directed medical therapy is improving. Seasonal patterns may play a pivotal role, biasing towards a larger and unhealthier population earlier in the year. Our cohort, as with prior seasonal analyses, exhibited a significant leftward skew in monthly admission volume (Supplementary material online, *Figure S3*).^21,22^ These longitudinal trends may be amplified by regression to the mean: more extreme readmission rates earlier in the year will decline over time and be included in the randomly sampled population.^23,24^

When including multiple index admissions, no blanking as opposed to blanking increased readmission rates (adjusted model difference of 2.3% for calendar year in both HF-specific and all-cause rates). In the United States, these opposing approaches are used by the AHRQ and CMS, respectively,^5^ and both have been used in observational research examining 30-day readmission rates.^13,25,26^ CMS specifically addresses unplanned readmissions. In the CMS measure, if the first readmission after discharge is planned, any subsequent unplanned readmission is not counted as part of the index admission.^5^ This is because the unplanned readmission might be linked to care provided during the intervening planned readmission rather than during the initial admission.^5^ In contrast, AHQR assesses the percentage of admissions with a readmission within 30 days without diagnostic exclusions, which includes both planned and unplanned readmissions.^3^ Therefore, blanking the first 30 days after index admission aims to exclude planned readmissions and avoid penalizing healthcare providers for planned readmissions they cannot control. This creates a more equitable measure of unplanned readmissions, which more accurately reflects the quality of care during the index admission.^5^ However, it may underestimate the true readmission rate.

### International Classification of Diseases codes

Readmission rates were higher when the index hospitalization and associated readmissions were both defined using narrow vs. broad ICD-10 code sets (adjusted model, range 0.3–0.9%). Broader definitions are used by the UK National HF Audit (I11.×, I25.×, I42.×, I50.×)^7^ and US CMS (I09.×, I11.×, I13.×, I50.×),^5^ while the Canadian HF Quality Indicators group recently restricted to only I50.×.^12^ Narrow definitions typically reduce sensitivity but increase specificity and positive predictive value.^11^ The identified index admissions will therefore have a greater proportion of patients with HF (i.e. fewer false positives) and corresponding higher readmission rate. In addition to altering the proportion of the index population with HF, different codes may potentially identify patients with different characteristics and disease severity. These considerations must be balanced against the need for sensitive definitions to minimize missing HF cases with failures of care.^11^

### Implications

A recent meta-analysis compared global 30-day hospital readmission rates for patients with HF.^10^ There was substantial variation both globally and within continents, ranging from 2% to 28%. The observed variability in readmission rates was not associated with the countries’ economic metrics, health care expenditure, or the competing risk of mortality. Our analysis raises the possibility that variations in how readmissions are defined may contribute to this heterogeneity. We advocate for a standardized approach to defining HF readmissions to ensure transparent, reproducible, and comparable reporting. This would preferably include multiple consecutive admissions with no blanking period after an index admission defined using broad ICD-10 codes, coupled with a process to exclude planned readmissions. This would minimize selection bias, enhance generalizability, accurately represent healthcare resource utilization, and ensure high-risk groups are not deprioritized in reporting. A broad definition will also likely be less sensitive to local differences in practice compared with more selective processes (e.g. 30-day blanking, narrower ICD-10 codes) and therefore facilitate national and international comparisons. Future reporting and research should ensure clear reporting of definitions, including all factors examined in our analysis.

### Strengths and limitations

Our study includes a large sample of consecutive HF hospitalizations from multiple centres over 3 years, adjudicated by trained chart abstractors. The government-funded universal healthcare and linkage to the provincial hospitalization database ensure comprehensive ascertainment of readmissions to any hospital, including those outside the audited hospitals. Although the results are likely generalizable to other healthcare systems, the magnitude of each factor's influence may vary in different cohorts. While we suggest a broad inclusive definition is desirable, the implications of this relative to other definitions warrant investigation in terms of population characteristics, service planning, economic policy, and health system outcomes.

## Conclusions

Calculated 30-day readmission rate after HF hospitalization varied more than two-fold when applying different approaches. Consistent methods are needed to calculate 30-day readmission rates to ensure transparent, reproducible, and comparable reporting. Internationally harmonized data standards are required not just for data collection but also the associated performance metrics.^27^

## Supplementary Material

qcae026_Supplemental_File

## Data availability

The data underlying this article cannot be shared publicly due to the privacy of individuals that participated in the study.
