# Supplementary material for: Impact of the method of calculating 30-day readmission rate after hospitalization for heart failure. Data from the VancOuver CoastAL Acute Heart Failure (VOCAL-AHF) registry
Source: Eur Heart J Qual Care Clin Outcomes. 2024 Apr 12;10(6):523–30. doi: 10.1093/ehjqcco/qcae026 (PMC11398898; doi:10.1093/ehjqcco/qcae026)
Supplement: qcae026_Supplemental_File [file qcae026_supplemental_file.docx]

**Supplementary Equation 1.** Readmission rate calculation using different index selection methods.

|  | 1. Multiple index admission (No-blanking):   $RR=\frac{\sum_{i=1}^{N} \sum_{t=1}^{\left\vert T_{i} \right\vert} 1_{\left\{ n_{tik}>0 \right\}}}{\sum_{i=1}^{N} \left\vert T_{i} \right\vert}$ |
| --- | --- |
|  | 1. Multiple index admission (Blanking):   $RR=\frac{\sum_{i=1}^{N} \sum_{t=1}^{\left\vert S_{i} \right\vert} 1_{\left\{ n_{tik}>0 \right\}}}{\sum_{i=1}^{N} \left\vert S_{i} \right\vert}$ |
| $RR:$ | readmission rate |
| $t:$ | index. |
| $i:$ | individual patient $i$. |
| $k:$ | duration of interval ($k=0$ implies 30 days, $k=1$ implies 31 days). |
| $n_{tik}:$ | the number of readmissions in interval $k$ after index $t$ for patient $i$. |
| $T_{i}:$ | the set of index admissions for patient $i \left( t\in T_{i} \right)$. |
| $S_{i}$ | $S_{i}=\left\{ t\in T_{i}\vert\text{date}_{t}-\text{date}_{t-1}>k \text{interval} \right\}, S_{i}\subset T_{i}$ |
| $\left\vert T_{i} \right\vert$ | total number of indices for patient $i$ |

Note: If a single index is considered for rate calculation: $\left| T_{i} \right|=1, \left| S_{i} \right|=1$

**Supplementary Table 1.** Different index admission selection methods employed by organizations and research publications in reporting the 30-day readmission rate.

|  | **Index Admission Selection Method** |
| --- | --- |
| **Organizations*** |  |
| UK National HF Audit | Single First-in-year |
| Canadian Institute for Health Information (CIHI) | Multiple |
| Centers for Medicare and Medicaid Services (CMS) | Multiple Blanking |
| 3M^TM^ Potentially Preventable Readmissions | Multiple Blanking |
| Healthcare Research and Quality (AHRQ) | Multiple No-Blanking |
| UnitedHealth Group | Multiple No-Blanking |
| **Heart failure and cardiology research** |  |
| Witcraft 21^1^ (including HF) | Single First-in-year |
| Kwok 21^2^ (HF) | Single First-in-year |
| Lawson 21^3^ (HF) | Single First-in-year |
| Samsky 19^4^ (HF) | Single First-in-year |
| Mcalister 17^5^ (HF) | Single First-in-year |
| Xiao 18^6^ (including HF) | Single First-in-year |
| Zepeda 19^7^ (HF) | Single First-in-year |
| Chung 17^8^ (HF) | Single First-in-year |
| Hummel 14^9^ (HF) | Single Random sampling |
| Davis 22^10^ (including HF) | Single Random sampling |
| Hubbard 14^11^ (AF) | Single Random sampling |
| Tellini 15^12^ (HF) | Multiple Blanking |
| Friebel 18^13^ (HF) | Multiple Blanking |
| Nasir 10^14^ (HF) | Multiple Blanking |
| Salsabili 20^15^ (AF) | Multiple No-Blanking |
| William 18^16^ (MI) | Multiple No-Blanking |

Abbreviations: AF, Atrial fibrillation; HF, Heart failure; MI, Myocardial infarction.

* For organizations’ references, refer to the article.

**Supplementary Table 2.** Absolute HF specific readmission rates according to different definitions.

| ICD code | Index selection  method | Survival adjustment | Index  day | Reference period | Readmission rate (%) |
| --- | --- | --- | --- | --- | --- |
| 0 | First-in-year | 0 | 0 | 0 | 10.5 |
| 0 | First-in-year | 0 | 0 | 1 | 9.9 |
| 0 | First-in-year | 0 | 1 | 0 | 10.6 |
| 0 | First-in-year | 0 | 1 | 1 | 10.1 |
| 0 | First-in-year | 1 | 0 | 0 | 10.6 |
| 0 | First-in-year | 1 | 0 | 1 | 10.1 |
| 0 | First-in-year | 1 | 1 | 0 | 10.7 |
| 0 | First-in-year | 1 | 1 | 1 | 10.3 |
| 0 | Random Sampling | 0 | 0 | 0 | 7.0 |
| 0 | Random Sampling | 0 | 0 | 1 | 6.5 |
| 0 | Random Sampling | 0 | 1 | 0 | 7.0 |
| 0 | Random Sampling | 0 | 1 | 1 | 6.7 |
| 0 | Random Sampling | 1 | 0 | 0 | 7.2 |
| 0 | Random Sampling | 1 | 0 | 1 | 6.7 |
| 0 | Random Sampling | 1 | 1 | 0 | 7.2 |
| 0 | Random Sampling | 1 | 1 | 1 | 6.8 |
| 0 | Blanking | 0 | 0 | 0 | 11.9 |
| 0 | Blanking | 0 | 0 | 1 | 11.1 |
| 0 | Blanking | 0 | 1 | 0 | 12.0 |
| 0 | Blanking | 0 | 1 | 1 | 11.4 |
| 0 | Blanking | 1 | 0 | 0 | 12.1 |
| 0 | Blanking | 1 | 0 | 1 | 11.3 |
| 0 | Blanking | 1 | 1 | 0 | 12.3 |
| 0 | Blanking | 1 | 1 | 1 | 11.6 |
| 0 | No-blanking | 0 | 0 | 0 | 14.1 |
| 0 | No-blanking | 0 | 0 | 1 | 12.9 |
| 0 | No-blanking | 0 | 1 | 0 | 14.3 |
| 0 | No-blanking | 0 | 1 | 1 | 13.2 |
| 0 | No-blanking | 1 | 0 | 0 | 14.5 |
| 0 | No-blanking | 1 | 0 | 1 | 13.2 |
| 0 | No-blanking | 1 | 1 | 0 | 14.7 |
| 0 | No-blanking | 1 | 1 | 1 | 13.5 |
| 1 | First-in-year | 0 | 0 | 0 | 10.9 |
| 1 | First-in-year | 0 | 0 | 1 | 10.3 |
| 1 | First-in-year | 0 | 1 | 0 | 11.1 |
| 1 | First-in-year | 0 | 1 | 1 | 10.5 |
| 1 | First-in-year | 1 | 0 | 0 | 11.1 |
| 1 | First-in-year | 1 | 0 | 1 | 10.5 |
| 1 | First-in-year | 1 | 1 | 0 | 11.3 |
| 1 | First-in-year | 1 | 1 | 1 | 10.7 |
| 1 | Random Sampling | 0 | 0 | 0 | 7.1 |
| 1 | Random Sampling | 0 | 0 | 1 | 7.3 |
| 1 | Random Sampling | 0 | 1 | 0 | 7.2 |
| 1 | Random Sampling | 0 | 1 | 1 | 7.5 |
| 1 | Random Sampling | 1 | 0 | 0 | 7.3 |
| 1 | Random Sampling | 1 | 0 | 1 | 7.5 |
| 1 | Random Sampling | 1 | 1 | 0 | 7.4 |
| 1 | Random Sampling | 1 | 1 | 1 | 7.7 |
| 1 | Blanking | 0 | 0 | 0 | 12.2 |
| 1 | Blanking | 0 | 0 | 1 | 11.4 |
| 1 | Blanking | 0 | 1 | 0 | 12.3 |
| 1 | Blanking | 0 | 1 | 1 | 11.6 |
| 1 | Blanking | 1 | 0 | 0 | 12.4 |
| 1 | Blanking | 1 | 0 | 1 | 11.6 |
| 1 | Blanking | 1 | 1 | 0 | 12.6 |
| 1 | Blanking | 1 | 1 | 1 | 11.9 |
| 1 | No-blanking | 0 | 0 | 0 | 14.4 |
| 1 | No-blanking | 0 | 0 | 1 | 13.1 |
| 1 | No-blanking | 0 | 1 | 0 | 14.6 |
| 1 | No-blanking | 0 | 1 | 1 | 13.3 |
| 1 | No-blanking | 1 | 0 | 0 | 14.8 |
| 1 | No-blanking | 1 | 0 | 1 | 13.5 |
| 1 | No-blanking | 1 | 1 | 0 | 15.0 |
| 1 | No-blanking | 1 | 1 | 1 | 13.7 |

Refer to the article’s Table 1 for the detailed description of each factor and category definition. ICD-10 code (HF-specific): broad 0 vs narrow 1; survival adjustment: survived at discharge 0 vs survived at 30-days 1; index day: discharge day 0 vs day after discharge 1; reference period: calendar year 0 vs fiscal year 1.

**Supplementary Table 3.** Comparison of baseline characteristics between patients with a single admission during the year (no history of readmission in a year) and those with multiple HF-admissions.

|  | One admission per year  N = 1,357 | >1 admission per year  N = 478 | p-value |
| --- | --- | --- | --- |
| Age | 73±15 | 75±15 | 0.07 |
| Sex (female) | 575 (42%) | 213 (45%) | 0.4 |
| Chronic kidney disease | 428 (32%) | 205 (43%) | <0.001 |
| Chronic obstructive pulmonary disease | 250 (18%) | 104 (22%) | 0.1 |
| Asthma | 80 (5.9%) | 27 (5.6%) | 0.8 |
| Malignancy | 200 (15%) | 72 (15%) | 0.9 |
| Liver disease | 75 (5.5%) | 47 (9.8%) | 0.001 |
| Anemia | 220 (16%) | 111 (23%) | <0.001 |
| Peptic ulcer disease | 68 (5.0%) | 25 (5.2%) | 0.9 |
| Dementia | 93 (6.9%) | 28 (5.9%) | 0.5 |
| Myocardial infarction | 279 (21%) | 122 (26%) | 0.02 |
| Cerebrovascular accident | 150 (11%) | 56 (12%) | 0.7 |
| Transient ischemic attack | 67 (4.9%) | 20 (4.2%) | 0.5 |
| Peripheral artery disease | 87 (6.4%) | 40 (8.4%) | 0.2 |
| Diabetes mellitus | 480 (35%) | 213 (45%) | <0.001 |
| Hypertension | 963 (71%) | 362 (76%) | 0.04 |
| Atrial fibrillation | 683 (50%) | 288 (60%) | <0.001 |
| Venous thromboembolism | 74 (5.5%) | 36 (7.5%) | 0.1 |
| Previous HF diagnosis | 640 (47%) | 313 (65%) | <0.001 |
| HF hospitalization | 596 (44%) | 298 (62%) | <0.001 |
| Heart transplant | 3 (0.2%) | 3 (0.6%) | 0.2 |

Values indicated as mean ± SD or n (%)

P-value determined using Wilcoxon rank sum test, Pearson's Chi-squared test or Fisher's exact test as appropriate.

**Supplementary Table 4.** Absolute all-cause readmission rates according to different definitions.

| ICD code | Index selection method | Survival adjustment | Index day | Reference period | Readmission rate (%) |
| --- | --- | --- | --- | --- | --- |
| 0 | First-in-year | 0 | 0 | 0 | 20.6 |
| 0 | First-in-year | 0 | 0 | 1 | 19.6 |
| 0 | First-in-year | 0 | 1 | 0 | 21.1 |
| 0 | First-in-year | 0 | 1 | 1 | 20.2 |
| 0 | First-in-year | 1 | 0 | 0 | 20.8 |
| 0 | First-in-year | 1 | 0 | 1 | 19.9 |
| 0 | First-in-year | 1 | 1 | 0 | 21.4 |
| 0 | First-in-year | 1 | 1 | 1 | 20.5 |
| 0 | Random Sampling | 0 | 0 | 0 | 20.6 |
| 0 | Random Sampling | 0 | 0 | 1 | 19.9 |
| 0 | Random Sampling | 0 | 1 | 0 | 21.0 |
| 0 | Random Sampling | 0 | 1 | 1 | 20.3 |
| 0 | Random Sampling | 1 | 0 | 0 | 19.6 |
| 0 | Random Sampling | 1 | 0 | 1 | 18.8 |
| 0 | Random Sampling | 1 | 1 | 0 | 19.8 |
| 0 | Random Sampling | 1 | 1 | 1 | 19.0 |
| 0 | Blanking | 0 | 0 | 0 | 25.4 |
| 0 | Blanking | 0 | 0 | 1 | 24.0 |
| 0 | Blanking | 0 | 1 | 0 | 25.9 |
| 0 | Blanking | 0 | 1 | 1 | 24.6 |
| 0 | Blanking | 1 | 0 | 0 | 25.9 |
| 0 | Blanking | 1 | 0 | 1 | 24.4 |
| 0 | Blanking | 1 | 1 | 0 | 26.5 |
| 0 | Blanking | 1 | 1 | 1 | 25.1 |
| 0 | No-blanking | 0 | 0 | 0 | 27.6 |
| 0 | No-blanking | 0 | 0 | 1 | 26.1 |
| 0 | No-blanking | 0 | 1 | 0 | 28.2 |
| 0 | No-blanking | 0 | 1 | 1 | 26.7 |
| 0 | No-blanking | 1 | 0 | 0 | 28.4 |
| 0 | No-blanking | 1 | 0 | 1 | 26.8 |
| 0 | No-blanking | 1 | 1 | 0 | 28.9 |
| 0 | No-blanking | 1 | 1 | 1 | 27.4 |
| 1 | First-in-year | 0 | 0 | 0 | 21.6 |
| 1 | First-in-year | 0 | 0 | 1 | 20.4 |
| 1 | First-in-year | 0 | 1 | 0 | 22.1 |
| 1 | First-in-year | 0 | 1 | 1 | 21.0 |
| 1 | First-in-year | 1 | 0 | 0 | 21.9 |
| 1 | First-in-year | 1 | 0 | 1 | 20.8 |
| 1 | First-in-year | 1 | 1 | 0 | 22.5 |
| 1 | First-in-year | 1 | 1 | 1 | 21.4 |
| 1 | Random Sampling | 0 | 0 | 0 | 22.1 |
| 1 | Random Sampling | 0 | 0 | 1 | 21.3 |
| 1 | Random Sampling | 0 | 1 | 0 | 22.7 |
| 1 | Random Sampling | 0 | 1 | 1 | 21.8 |
| 1 | Random Sampling | 1 | 0 | 0 | 21.1 |
| 1 | Random Sampling | 1 | 0 | 1 | 19.9 |
| 1 | Random Sampling | 1 | 1 | 0 | 21.4 |
| 1 | Random Sampling | 1 | 1 | 1 | 20.3 |
| 1 | Blanking | 0 | 0 | 0 | 26.4 |
| 1 | Blanking | 0 | 0 | 1 | 24.8 |
| 1 | Blanking | 0 | 1 | 0 | 27.0 |
| 1 | Blanking | 0 | 1 | 1 | 25.3 |
| 1 | Blanking | 1 | 0 | 0 | 26.9 |
| 1 | Blanking | 1 | 0 | 1 | 25.3 |
| 1 | Blanking | 1 | 1 | 0 | 27.6 |
| 1 | Blanking | 1 | 1 | 1 | 25.9 |
| 1 | No-blanking | 0 | 0 | 0 | 28.6 |
| 1 | No-blanking | 0 | 0 | 1 | 26.9 |
| 1 | No-blanking | 0 | 1 | 0 | 29.1 |
| 1 | No-blanking | 0 | 1 | 1 | 27.4 |
| 1 | No-blanking | 1 | 0 | 0 | 29.4 |
| 1 | No-blanking | 1 | 0 | 1 | 27.7 |
| 1 | No-blanking | 1 | 1 | 0 | 29.9 |
| 1 | No-blanking | 1 | 1 | 1 | 28.2 |

**Supplementary Figure 1.** A hypothetical patient readmission chain diagram in a specific year, illustrating two different methods in defining index admission and readmissions when multiple index admission per patient is used in the calculation of readmission rates. Each set of admission and discharge is illustrated in separate boxes respectively. As illustrated, 5 index admissions and 4 readmissions are specified within 30-days, based on no-blanking approach (upper part of the diagram). The respective numbers for blanking approach (lower part of the diagram) are 1 index and 4 readmissions within 30-days.


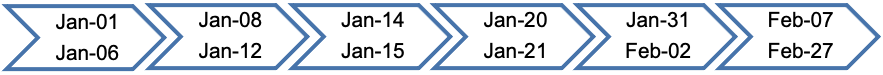


Multiple index admission using no-blanking approach

Multiple index admission definition using blanking approach

**Supplementary Figure 2.** Recreation of Figure 2 from the article, with standardized y-axes for all three sub-figures. Absolute readmission rate by index admission method categories for all-cause, HF-readmissions, and non-HF readmissions (from top to bottom respectively).

**
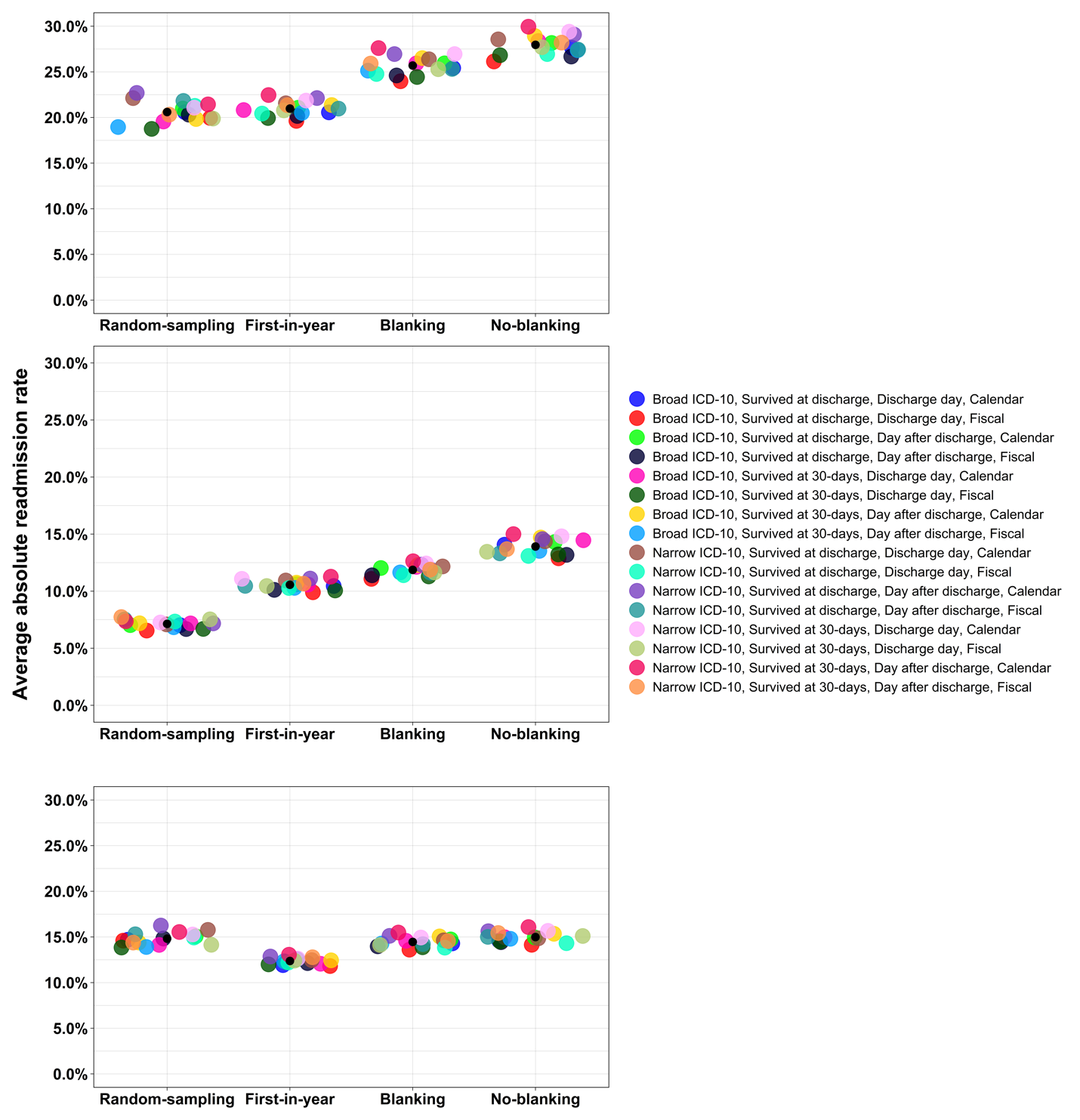
**

**Supplementary Figure 3.** Seasonal variation in average proportion of index admissions over 3 years (2016-2018). **A.** Index admission defined using first-in-year method **B.** Index admission defined using random sampling.

**
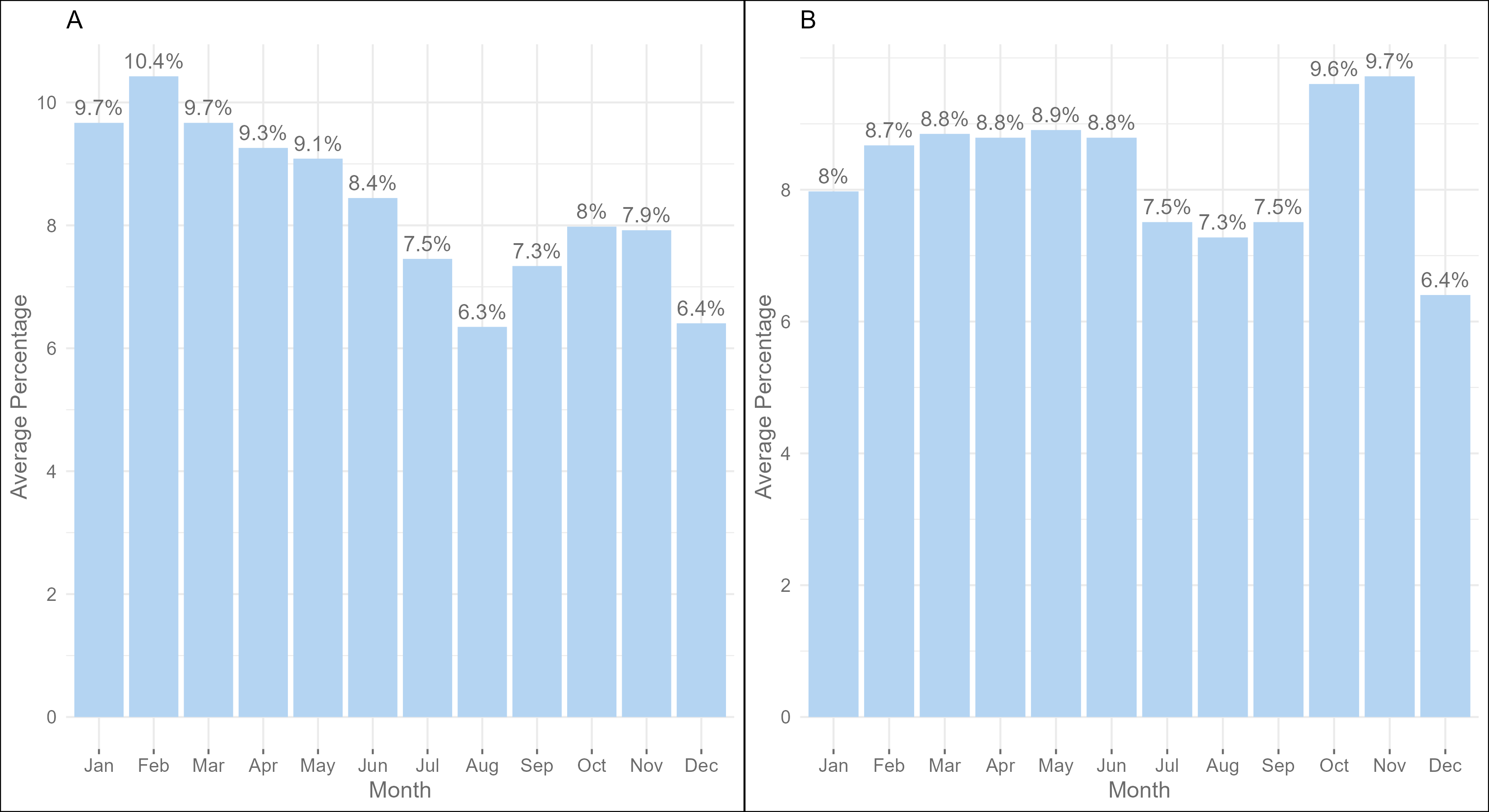
**

References

1. Witcraft EJ, Norris AM, Fudzie SS, Vest M-H, Johnson N, Rush J, et al.. Impact of medication bedside delivery program on hospital readmission rates. J Am Pharm Assoc 2021;**61**(1):95-100. e1.

2. Kwok CS, Abramov D, Parwani P, Ghosh RK, Kittleson M, Ahmad FZ, et al. Cost of inpatient heart failure care and 30-day readmissions in the United States. Int J Cardiol 2021;**329**:115-122.

3. Lawson C, Crothers H, Remsing S, Squire I, Zaccardi F, Davies M, et al. Trends in 30-day readmissions following hospitalisation for heart failure by sex, socioeconomic status and ethnicity. EClinicalMedicine 2021;**38**.

4. Samsky MD, Ambrosy AP, Youngson E, Liang L, Kaul P, Hernandez AF, et al. Trends in readmissions and length of stay for patients hospitalized with heart failure in Canada and the United States. JAMA Cardiol 2019;**4**(5):444-453.

5. McAlister FA, Youngson E, Kaul P. Patients with heart failure readmitted to the original hospital have better outcomes than those readmitted elsewhere. J Am Heart Assoc 2017;**6**(5):e004892.

6. Xiao R, Miller JA, Zafirau WJ, Gorodeski EZ, Young JB. Impact of home health care on health care resource utilization following hospital discharge: a cohort study. Am J Med 2018;**131**(4):395-407. e35.

7. Zepeda I, Li DL, Quispe R, Taub CC. Clinical characteristics of young patients with heart failure with reduced ejection fraction in a racially diverse cohort. Crit Pathw Cardiol 2019;**18**(2):80.

8. Chung J, Noh E, Gwak H. Evaluation of the predictors of readmission in Korean patients with heart failure. J Clin Pharm Ther 2017;**42**(1):51-57.

9. Hummel SL, Katrapati P, Gillespie BW, DeFranco AC, Koelling TM. Impact of prior admissions on 30-day readmissions in medicare heart failure inpatients. In: *Mayo Clinic Proceedings*. *2014*: Abstract 89, p. 623-630. Elsevier.

10. Davis S, Zhang J, Lee I, Rezaei M, Greiner R, McAlister FA, et al. Effective hospital readmission prediction models using machine-learned features. BMC Health Serv Res 2022;**22**(1):1415.

11. Hubbard M, Frost S, Siu K, Quon N, Esposito D. Association between outpatient visits following hospital discharge and readmissions among Medicare beneficiaries with atrial fibrillation and other chronic conditions. Am J Med Qual 2014;**29**(3):206-212.

12. Tellini M, Petrioli A, Forni S, Morettini A. The revolving door syndrome in internal medicine: a study on 11,846 subjects discharged from all Internal Medicine Departments of Tuscany with diagnosis of heart failure and pneumonia. Ital J Med 2015;**9**(2):150-156.

13. Friebel R, Hauck K, Aylin P, Steventon A. National trends in emergency readmission rates: a longitudinal analysis of administrative data for England between 2006 and 2016. BMJ open 2018;**8**(3):e020325.

14. Nasir K, Lin Z, Bueno H, Normand S-LT, Drye EE, Keenan PS, et al. Is same-hospital readmission rate a good surrogate for all-hospital readmission rate? Med Care 2010;**48**(5):477-481.

15. Salsabili M. Atrial Fibrillation Readmissions: Temporal Trends, Risk Factors and Data Driven Modeling. University of Minnesota 2021.

16. Williams JH, Jarosek S, Carroll N, Fan Y, Hall AG. Health system affiliation and 30-day readmission after heart attack in black men. Am J Prev Med 2018;**55**(5):S22-S30.
